# Effects of dietary extrusion on rumen fermentation, nutrient digestibility, performance and milk composition of dairy cattle: a meta-analysis

**DOI:** 10.5713/ab.23.0012

**Published:** 2023-05-04

**Authors:** Sazli Tutur Risyahadi, Rima Shidqiyya Hidayati Martin, Novia Qomariyah, Suryahadi Suryahadi, Heri Ahmad Sukria, Anuraga Jayanegara

**Affiliations:** 1 Department of Nutrition and Feed Technology, Faculty of Animal Science, IPB University, Bogor 16680, Indonesia; 2 Animal Feed and Nutrition Modelling (AFENUE) Research Group, Department of Nutrition and Feed Technology, Faculty of Animal Science, IPB University, Bogor 16680, Indonesia; 3 Center of Tropical Animal Studies (Centras), IPB University, Bogor 16153, Indonesia; 4 South Sulawesi Assessment Institute of Agriculture Technology, Makassar 90242, Indonesia; 5 Faculty of Science and Technology, Pahlawan Tuanku Tambusai University, Kampar, Riau 28412, Indonesia

**Keywords:** Dairy Cattle, Extrusion, Meta-analysis, Performance, Ruminal Fermentation

## Abstract

**Objective:**

The present study aimed to evaluate the effects of extruded and unextruded feeding on the performance, milk composition, digestibility and ruminal fermentation of dairy cows through a meta-analysis.

**Methods:**

The database was compiled from 53 studies in Scopus and PubMed. The data were analyzed using a random effects model in OpenMEE software. Extruded feed was grouped as the experiment group while and the others as control group. The bias of publication in the main parameter of dairy performance was evaluated by a funnel plot.

**Results:**

The result showed that extruded feed enhanced the milk yield, dry matter and crude protein digestibility, butyrate and valerate acid production (p<0.05). Meanwhile, the extruded feed significantly decreased the milk fat and protein concentration (p<0.05). Also, the iso-butyrate and iso-valerate in unextruded feeding was significantly higher than the extruded feed (p<0.05).

**Conclusion:**

It was concluded from the meta-analysis that extruded feed effectively improved the milk production and milk lactose concentration, dry matter and protein digestibility, but not the milk fat and protein concentration.

## INTRODUCTION

Grains are used as either energy or protein sources for dairy animals. Protein source from grains is desirable because of its optimal amino acid profile, high digestibility and palatability while cereal grains contain high metabolizable energy. Nevertheless, several grains contain anti-nutrients which can reduce availability of nutrient and hamper animal performance [1,2]. Generally, various studies confirm that grain processing is required to minimize the amount of the anti-nutritional factors and some grains i.e., treated corn and sorghum are more resistant to rumen fermentation [3,4]. One of the techniques uses heat processing such as extrusion. This process has been associated with increased efficiency of fermentation by altering the protein matrix of the endosperm and the starch structure, thus allowing a better utilization by microbial enzymatic digestion. Yet, the consequence of extrusion process is protein denaturation which occurs in protein because of its highly reactive functional groups [5]. Therefore, extrusion cooking may additionally change molecular systems, weight and size of proteins which may be result in changes to crude protein (CP) sub-fractions.

Extrusion of grain involves using moisture, high temperature and pressure to reach a high stage of starch gelatinization. Also, those modifications might also have an effect on the palatability and standard the feeding price of the final product for ruminant feed. Temperature and heating duration must be carefully controlled for grains with high protein to optimize the digestible protein content and prevent the increasement of undigestible protein fraction or heat damage. Furthermore, the overprotection of protein can occur if the temperature is too high and thereby reducing the intestinal digestibility [6]. It has previously been shown that extruded feeding supplements reduced methane production and yield of lactating dairy cows, but dry matter intake (DMI) and milk yield have been additionally reduced [7,8].

Though many experiments have reported the effect of extruded grains in dairy performance and digestibility, there is no work to summarize those research results quantitatively and a meta-analysis of the effect of extruded feeding on dairy cows has not been conducted. This indicates that further study of extrusion on grain or feed in term of enhancing dairy cows’ performance and production is needed. The quantitative evaluation of input (unextruded and extruded feed) and output (performance, milk quality, digestibility and ruminal fermentation) by random effect meta-analysis method statistical approach might allow for assessing the relationships. Thus, the objective was to evaluate the effects of both extruded and unextruded feed on the dairy cow performance, milk composition, digestibility and ruminal fermentation by using meta-analysis method.

## MATERIALS AND METHODS

### Literature search and selection criteria

A database was developed from several types of literature which reported the utilization of extruded feed on dairy cow. The searching of literature was conducted using Scopus and PubMed and the keywords used were ‘extruded’, ‘dairy’, and ‘cow/cattle’. The database was made in December 2022 from the Scopus research database while the PubMed research database was built up in January 2023. The initial search resulted in 359 articles with the selection criteria were: i) English-language articles; ii) direct comparison between extruded and unextruded feed; iii) the studies were conducted on dairy cattle; iv) comparison on animal performance, milk composition, digestibility and ruminal fermentation and; v) replication and variance were reported (standard deviation or standard error of the means). These criteria followed the Preferred Reporting Item for Systematic Reviews and Meta-Analysis (PRISMA) protocol.

**Figure 1:**
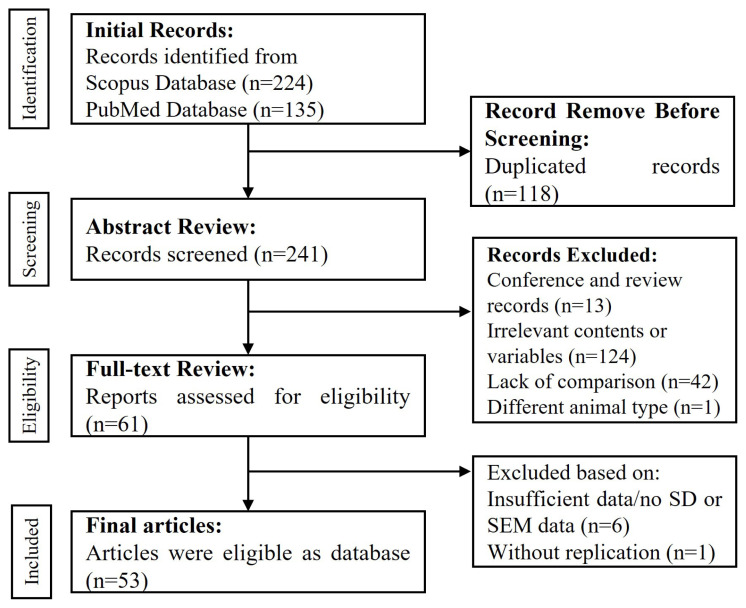
Flow chart of articles selection process based on Preferred Reporting Item for Systematic Reviews and Meta-Analysis (PRISMA) protocol.

The selection process is shown in Figure 1. Concisely, the initial search was screened based on the title. A total of 14 articles was excluded for several reasons (non-related title, review articles or conference proceedings). In abstract screening, the useful literature consisted of 241 articles and the rest were excluded because duplication, irrelevant contents or variables and animal type. Hence, the full text evaluation resulted in 61 articles while 180 articles were excluded due to lack comparison (n = 42), conference and review articles (n = 13), irrelevant contents or variables (n = 124) and different animal type (n = 1). The final articles (n = 53) after assessment consider as database in meta-analysis (Table 1).

A fail-safe number (*N*ft) was intended to recognize the publication bias caused by the insignificant studies which were not included on the analysis. *N*ft > 5N + 10 was considered to provide evidence of a robust meta-analysis model. *N*ft was calculated using Rosenthal et al’s method. The least sample size from individual studies was applied as *N*. Moreover, funnel plot was conducted to assess the publication bias.

### Database development

The bibliography, animal breed, body weight (BW), age, extrusion process and feed ingredients were inputted to Microsoft Excel spreadsheet. Response variables included in database consisted of four groups, i.e., dairy performances (DMI), BW, body condition scoring (BCS), milk yield, and 4% fat-corrected milk (FCM) yield, milk composition (milk lactose concentration, milk fat concentration, and milk protein concentration), digestibility (dry matter digestibility [DMD], organic matter digestibility, crude protein digestibility [CPD], neutral detergent fiber [NDF] digestibility, and acid detergent fiber [ADF] digestibility) and ruminal fermentation (total volatile fatty acid [VFA], acetate acid, propionate acid, butyrate acid, iso-butyrate acid, iso-valerate, and valerate). The entire variables were converted into the same units of measurements. The descriptive statistics of database is presented in Table 2.

### Data analysis

All data were analyzed using the random effects meta-analysis method. The calculation was based on the standardized mean difference of Hedges’ where the mean value of unextruded feed was grouped into control group (X^C^) and the mean value of extruded feed was the experimental group (X^E^). The calculation as followed:


$$d=\frac{\left(X^{E}-X^{C}\right)}{S}J$$

*J* was the correction factor for the small sample size while *S* was the pooled standard deviation. The equation was derived from Sánchez-Meca and Marín-Martínez [9] with 95% confidence interval. The data were analyzed by using OpenMEE application for dairy performance (5 items), milk composition (3 items), digestibility (5 items) and ruminal fermentation (7 items).

## RESULTS

**Figure 2:**
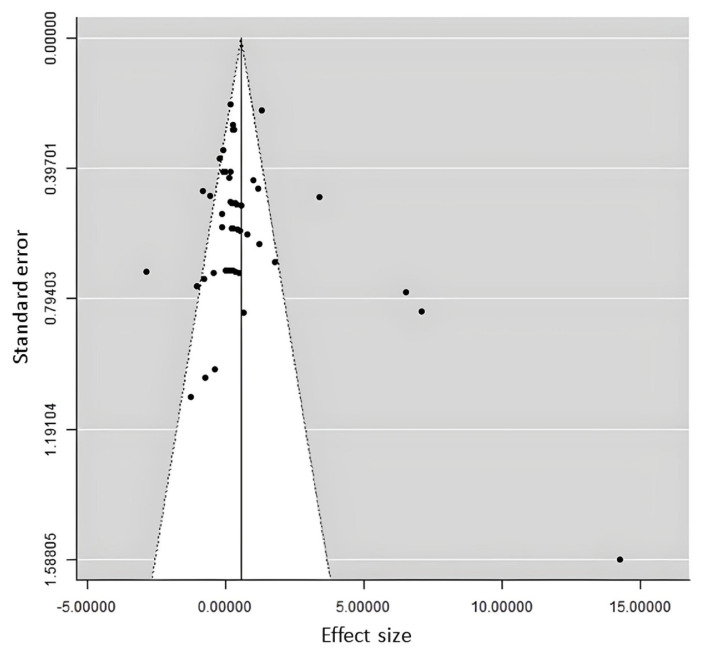
Funnel plot for milk yield.

Due to conflicting research findings and small sample size, not all results could be considered reliable due to publication bias. The funnel plot of milk yield as the main parameter in dairy performance showed in Figure 2. Briefly, the fail-safe number (Nfs) indicated which studies were suitable to be included into the final strong conclusions. This number expressed how many sample study sizes should be added in order to change the initial effect size into a negligible variable. If Nfs > 5N + 10, where N was the study effect size used to calculate the initial effect size, then the result could be considered as the final robust conclusion [10]. According to these fail-safe number rules, robust parameters of milk production included milk yield, milk fat concentration, milk protein concentration, milk lactose concentration, CPD, iso-butyrate and iso-valerate fatty acid.

This meta-analysis study used Q statistics test, τ^2^ and I^2^ to examine heterogeneity. The *Q*-statistic was the weighted sum of the squared values of each study effect size’s deviation from the mean effect size of all studies. The estimate of the population variable tau (τ) was the standard deviation of the overall effect size and τ^2^ represents the variance of the overall effect size. The I^2^ index was a measure of the proportion of unexplained heterogeneity. Based on Heterogeneity Q statistics test, τ^2^ and I^2^ showed that some variables were categorized in high heterogeneity and others was low heterogeneity. In terms of dairy performance, DMI and Milk yield had excess heterogeneity when Q was higher than degree of freedom (NC-1) meanwhile BW, BCS, and 4% were categorized in low or no excess heterogeneity when Q was lower than degree of freedom (NC-1). For milk composition and ruminal fermentation, all of variables were high heterogeneity. In term of digestibility only ADF digestibility had no excess heterogeneity. Heterogeneity was impacted by several factors the number of studies in the meta-analysis, how much the study effect sizes varied from each other (between studies variance) and how much variance existed in the observed effect size for each study (within-study variance) [11]. Heterogeneity of this study was high due to different type of ingredient, extrusion process parameter, dairy cow ages and the concentration of extruded feed. The differences of heat extrusion processes influenced quality of extruded feed so it affected the digestibility. Nutrient intake and total-tract apparent digestibility in dairy cows fed diets containing extruded soybean meals (SBM) produced by low and high temperature were different [12]. Different concentrations of extruded feed ingredients affected milk composition. Providing high doses of extruded linseed could also have negative effects on end product processing, such as generating important material losses during the churning stage [13].

A meta-analysis results are shown in Table 3 and 4. Table 3 shows the detailed meta-analysis results of dairy performance and milk composition according to Cohen’s methodology. In comparison to unextruded feed, feed extrusion significantly increased the milk production (p<0.05) while the other parameters were unaffected by extrusion cooking. On the other hand, extruded feed decreased DMI and 4% FCM yield percentage (p<0.05). For body weight and body score condition, no significant effect of the extruded process was observed. In term of milk composition, extrusion cooking significantly contributed to increasing the milk lactose composition (p<0.05). On contrary, the other compositions such as milk fat and milk protein were higher in unextruded feed (p<0.05).

Based on the ruminal fermentation, the meta-analysis results of digestibility and production of VFA of feed extrusion are presented in Table 4. It was indicated that dry matter and CPD was significantly higher for extruded feed (p<0.05). The digestion of organic matter and fibrous fraction (NDF and ADF) were not significantly affected by the extrusion process. Moreover, extrusion cooking also influenced the production of VFA. Butyrate and valerate production were enhanced in extruded feed (p<0.05) while the iso-butyrate and iso-valerate were significantly increased in unextruded feed (p<0.05). The production of total VFA, acetate and propionate for both control and experimental group were not significantly affected.

## DISCUSSION

### Effects on dairy performance and milk composition

Milk production was one of the dairy performances that was significantly affected. Increased in milk yield was also reported by Mendowski et al [6]. The reduction in DMI, but without an affect on milk production also reported by Kozerski et al [14], increasing starea in concentrate reduced daily DMI without affecting milk yield and 4% FCM yield. Contrary to reports that non protein nitrogen reduced DMI and consequently milk production [15,16]. When the partial replacement of SBM with urea did not affect milk production while maintaining DMI the maintenance needs and production of metabolizable proteins were mainly met by microbial protein synthesis [17]. The extruded feed material is very stably different in density and therefore ferments at different positions in the rumen and giving the duodenal starch a distinct appearance pattern. Income issues might be related to this size and physical shape of extruded pellets and use of smaller pellets may have solved the problem of extruded barley feed [18]. Due to the relative increase in the proportion of propionic acid, acetate content increased starch breakdown in the rumen [19]. A higher proportion of acetate than propionate causes milk production to increase, because acetate is a precursor to milk production. In addition, extruded feed lowers its density so the cows might digest it more slowly. Lower grain density is a possible explanation for the decrease in DMI [20].

Feeding an extruded ration affected the milk lactose concentration, milk fat concentration and milk protein concentration. Decreased milk lactose concentration, milk fat concentration and milk protein concentration were also reported by Mendowski et al [6]. Extruded feed would decrease the lipids supplied by blends, and in fact extrusion increased their availability in the rumen. Extruded affects the milk lactose concentration, milk fat concentration and milk protein concentration was also reported by Shabi et al [20]. When the cows were given ground corn feed it increased the frequency of eating but did not result in changes in milk protein or energy efficiency of milk, on the other hand if extruded feed was given, there was an increase in feeding frequency and a change in milk protein.

In addition, extruded increased the milk lactose concentration, milk fat concentration and milk protein concentration as reported by Nocek and Braund [21], Yang and Varga [22], and Chouinard et al [23]. This difference might be due to the influence of the temperature used in the extrusion process. In addition, heating oil produced reducing agents that were capable of capturing hydrogen ions and possibly inhibited methanogenesis in the rumen. Reduced methanogenesis spares other hydrogen ions for the production of propionate, which leads to milk fat depression [24]. Furthermore, extrusion of grains in high dietary concentrate fed to dairy cows affected the lactose percentage [25]. The lactation curve of lactose percentage showed a strict correlation with milk yield. In fact, the amount of absorbed water in the alveoli was determined by lactose and it affected the volume of produced milk [26]. This meta-analysis showed a linear result in which increased lactose concentration resulted in increased milk yield.

### Effects on digestibility and ruminal fermentation

Extrusion feed affected the dry matter and CPD. Higher DMD was also reported by Berenti et al [27] and Gonthier et al [28]. This might be due to the digestion of CP. Increased CPD due to extrusion was proven in this meta-analysis work. Grain heat processing changed functional properties of its proteins, which in turn altered protein digestibility. The complex protein was broken down into small proteins such as peptides and this form was easier to digest by the animal [29]. During high temperature extrusion cooking, there was a decreasing of stable protein structures, and consequently polypeptides and peptides would be more available and more hydrolysable by digestive enzymes [30,31]. Moreover, alteration in protein fractions, molecular structural make-up and molecular weights might lead to changes in rumen undegradable protein (RUP) and rumen degradable protein fractions in adult ruminants. Samadi and Yu [5] reported heating process could enhance intestinal digestibility of RUP so it could improve the feed efficiency. Moreover, extrusion reduced the anti-nutritional factor in grains, such as kunitz domain/protease inhibitors in soybean, so the availability of protein increased [32]. Yet, higher temperature of extrusion could lead an overprotection of diet protein and reduced available amino acid in the intestine [6] although extrusion has been shown to improve true N digestibility [33,34].

Over processing would result in protein denaturation and most likely transform the proteins to a more resistant structure and cross-linkages formation between amino acids and reducing sugars such as Maillard reaction could occur. The effect of processing method on protein quality of feed might be more important and easier to detect in young dairy calves than in older calves because of their less developed gastrointestinal tract [1]. Despite an increase of CP digestibility which tended to increase post ruminal flow of microbial non-ammonia nitrogen (NAN), this did not result in an enhancing in milk protein concentration, which would suggest that the greater CP digestibility and microbial NAN flow might not necessarily result in a greater AA supply to the mammary gland [35].

Extrusion cooking did not affect the total VFA, acetate and propionate, yet other VFA i.e., butyrate, valerate, iso-butyrate and valerate were impacted. Among VFA, valerate and butyrate have a greater role in papillae development in the rumen [36,37]. Moreover, absorption of VFA might be related with ruminal papillae surface area, which would allow greater VFA diffusion transport through rumen tissue. Difference in ruminal butyrate and valerate concentration might have influenced the blood flow rate, which was also related with VFA uptake [38–40]. In contrast, the iso-butyrate and iso-valerate were higher in unextruded feed. As a reverse form of butyrate and valerate, the iso-butyrate and iso-valerate production might be higher when the reverse form was lower.

## CONCLUSION

The current research revealed that extrusion of grain affected the milk production and digestion of dairy cattle. In term of milk composition, extrusion could increase the lactose concentration, still the fat and protein concentration were significantly lower. The extruded feed could increase the dry matter and crude protein digestibility. Production of butyrate and valerate was increasing in extrusion feed while the iso-butyrate and iso-valerate were higher in unextruded grains.

## Figures and Tables

**Table 1:** Articles included in the meta-analysis

No.	Reference	Experiment	Animal breed	Initial body weight (kg)	Age (d)	Extrusion heat (°C)	Feed ingredients
1.	[41]	*In vivo*	Holstein	NA	116.00±64.00 DIM	120	Linseed
2.	[42]	*In vivo*	Friesian	155.00 172.00 185.00	NA	90	Corn
3.	[43]	*In vivo* *In situ*	Holstein	NA	NA	NA	Soybean
4.	[44]	*In vivo* *In situ*	Friesian	NA	80.00±41.00 DIM	NA	Soybean
5.	[45]	*In situ*	Chilean Holstein	NA	NA	140	Dehulled lupin Pea
6.	[46]	*In vivo* *In situ*	Holstein	650.00±23.00	45.00 – 50.00 DIM	195	Whole horse bean
7.	[27]	*In vivo* *In situ*	Holstein	40.30±0.63	1 of birth	150±2	Soybean meal
8.	[47]	*In vivo*	Holstein	NA	NA	152	Whole cottonseed
9.	[48]	*In vivo* *In situ*	Holstein	616.00	91.00 DIM	NA	Pima cottonseed cake
10.	[49]	*In vivo*	Holstein	550.00	65.30 DIM	141	Soybean meal
		*In situ*			65.70 DIM		
11.	[50]	*In vivo*	Holstein	NA	43.00±23.00 DIM	150	Full-fat soybean
12.	[51]	*In vivo*	Holstein	672.00±54.00	213.00±40.00 DIM	NA	Linseed – wheat
13.	[23]	*In vivo* *In situ*	Holstein	NA	35.00±10.00 of postpartum	120 130 140	Soybean
14.	[35]	*In vivo*	Holstein	712.00±54.00	90.00±31.00 DIM	160	WDDGS – pea
		*In situ*				161	WDDGS – canola meal
15.	[52]	*In vivo*	Italian Holstein	NA	99.00±55.00 DIM	NA	Corn
16.	[53]	*In vivo*	Holstein	NA	NA	105	Sorghum
						118	Soybean
17.	[54]	*In vivo*	Holstein	644.00±40.00	NA	120	Linseed
18.	[55]	*In vivo* *In situ*	Holstein	548.10±66.90	74.00±12.00 DIM	NA	Canola seed
19.	[56]	*In vivo* *In situ*	Holstein – Friesian	610.00	90.00	142	Rapeseed
20.	[57]	*In vivo*	Holstein	648.00±46.00	61.00±4.00 DIM	NA	Linseed
			Montbeliardes	646.00±51.00	76.00±5.00 DIM		
21.	[58]	*In vivo*	Friesian	546.00	NA	NA	Pea
22.	[12]	*In vivo*	Holstein	650.00±54.70	141.00±31.00 DIM	149	Soybean meal
		*In situ*				171	
23.	[28]	*In vivo*	Holstein	595.00±32.00	225.00±17.00 DIM	155	Flaxseed
24.	[59]	*In vivo*	Holstein	595.00±32.00	225.00±17.00 DIM	155	Flaxseed
25.	[60]	*In vivo*	Holstein	NA	−15.00 of postpartum	149	Soybean
26.	[61]	*In vivo*	Holstein	565.00±54.00 631.00±74.60	21.00±3.00 of postpartum	160	Soybean meal
27.	[62]	*In vitro*	NA	NA	NA	149	Soybean
28.	[63]	*In vivo*	Holstein	565.00	21.00 of postpartum	NA	Soybean
29.	[64]	*In vivo*	Lithuanian Black-and-White	590.00±20.00	30.00±6.00 DIM	135 – 155	Faba bean
30.	[65]	*In vivo*	Danish Holstein	655.00±57.00	209.00±98.00 DIM	90	Wheat
						115	Wheat – Soybean meal Maize Maize – Soybean meal
31.	[8]	*In vivo*	Holstein	672.00±54.00	213.00±40.00 DIM	NA	Linseed
32.	[66]	*In vivo*	Italian Holstein	604.00±109.00	140.00±25.00 DIM	NA	Pea
33.	[6]	*In vivo*	Holstein	754.00±58.00	65.00±21.00 DIM	140	Faba bean – Linseed
						160	Lupin seed – Linseed
34.	[67]	*In vivo*	Holstein	690.00±29.00	96.00±27.00 DIM	140	Fava bean
35.	[68]	*In vivo*	Holstein	712.70±92.30	116.50±17.50 DIM	NA	Flaxseed – Pea Flaxseed – Fava bean (containing tannin)
36.	[69]	*In vivo*	Holstein	713.00±50.00	NA	155	Flaxseed
37.	[70]	*In vivo*	Holstein	450.00	NA	116 138 160	Whole soybean
38.	[71]	*In vivo*	Holstein	660.00±55.00	119.00±23.00 DIM	NA	Canola meal
		*In situ*		694.00±56.00	220.00±71.00 DIM		
39.	[72]	*In vivo*	Holstein	584.00±15.00	14.00 of postpartum	140	Pea
40.	[73]	*In vivo*	Holstein Brown Swiss	NA	NA	NA	Soybean
41.	[74]	*In vivo*	Holstein	NA	106.00±49.70 DIM	121	Corn
42.	[75]	*In vivo*	Holstein	534.00±52.00	104.00±5.00 DIM	NA	Soybean
43.	[76]	*In vivo*	Holstein	NA	91.00 DIM	NA	Soybean/soybean meal
44.	[77]	*In vivo*	Holstein	NA	121.00±5.00 DIM	NA	Soybean
		*In situ*			112.00±11.00 DIM		
45.	[20]	*In vivo* *In situ*	Holstein	NA	103.00±20.00 DIM	200	Corn
46.	[78]	*In situ*	Holstein	558.00±14.00	NA	140	Whole soybean Pea Lupin Soybean meal Whole soybean – maize
47.	[79]	*In vivo*	Holstein	575.00	112.00±15.00 DIM	130	Soybean meal
48.	[80]	*In vivo*	Holstein	NA	70.00 DIM	150	Soybean
49.	[81]	*In vivo*	Holstein	NA	189.00±57.00 DIM	NA	Soybean
			Brown Swiss		126.00±49.00 DIM		
50.	[82]	*In vivo* *In situ*	Chinese Holtein	596.00±19.00	150.00 DIM	NA	Soybean
51.	[83]	*In vivo*	Holstein	42.00±0.50	1 of birth	NA	Full-fat soybean
52.	[84]	*In vivo*	Holstein	NA	7 of birth	120	Corn Soybean
53.	[85]	*In vivo* *In vitro*	Holstein	NA	36.00 DIM	NA	Soybean

DIM, day in milk; WDDGS, wheat dried distillers’ grains with soluble.

**Table 2:** Descriptive statistics of database

Variables	Unit	NC	Mean		Min		Max		SD	
			
			Unextruded	Extruded	Unextruded	Extruded	Unextruded	Extruded	Unextruded	Extruded
Dairy performance										
Dry matter intake (DMI)	kg/d	84	16.37	16.21	0.98	0.93	28.10	29.10	1.42	1.45
Body weight (BW)	kg	50	389.32	390.66	22.00	41.60	728.00	741.00	30.64	29.89
Body condition score (BCS)	point	27	2.94	2.96	2.10	2.20	3.40	3.40	0.25	0.25
Milk yield	kg/d	51	31.72	32.47	20.20	18.00	45.90	44.40	4.77	4.76
4% FCM yield	kg/d	32	28.92	28.88	20.30	18.40	42.60	43.70	4.27	4.27
Milk composition										
Lactose concentration	g/kg	40	47.47	47.49	40.50	43.20	52.10	51.90	1.55	1.58
Fat concentration	g/kg	46	35.94	33.20	7.90	3.17	48.00	48.80	3.98	4.22
Protein concentration	g/kg	49	31.14	30.49	28.50	27.50	37.00	35.90	2.00	1.95
Digestibility										
Dry matter (DM)	kg/kg	42	0.60	0.61	0.23	0.13	0.76	0.83	0.05	0.05
Organic matter (OM)	kg/kg	52	0.62	0.63	0.12	0.19	0.77	0.79	0.05	0.05
Crude protein (CP)	kg/kg	52	0.49	0.55	0.02	0.15	0.98	0.97	0.04	0.04
Neutral detergent fiber (NDF)	kg/kg	30	0.49	0.49	0.08	0.09	0.55	0.54	0.03	0.03
Acid detergent fiber (ADF)	kg/kg	18	0.49	0.49	0.33	0.30	0.66	0.66	0.04	0.04
Ruminal fermentation										
Total volatile fatty acid (VFA)	mmol/L	33	99.44	101.51	68.30	67.84	174.00	163.00	10.92	9.37
Acetate^1)^		49	59.85	59.62	44.30	38.50	70.03	70.60	3.39	3.39
Propionate		49	24.61	24.51	2.65	2.61	60.37	60.50	4.67	4.67
Butyrate		47	11.11	11.72	1.21	1.11	15.10	18.70	2.12	2.06
Iso-butyrate		31	1.72	1.62	0.24	0.25	12.80	13.50	0.32	0.32
Iso-valerate		34	1.68	1.54	0.40	0.35	3.39	2.93	0.30	0.30
Valerate		34	1.81	2.41	0.78	0.80	3.65	9.73	0.43	0.43

SD, standard deviation.

1) Individual VFAs are percent (%) of total VFA.

**Table 3:** Effects of feed extrusion on dairy performance and milk composition

Variables	NC	Estimate	Lower bound	Upper bound	Std. error	p-value	τ^2^	Q	Het. p-value	I^2^
Dairy performance										
DMI	84	−0.124	−0.250	0.03	0.065	0.056	0.072	108.067	0.034	23.196
BW	50	0.110	−0.016	0.236	0.064	0.087	0.000	46.602	0.571	0.000
BCS	27	0.040	−0.141	0.221	0.092	0.664	0.000	18.724	0.848	0.000
Milk yield	51	0.559	0.213	0.904	0.176	0.020	1.227	326.015	<0.001	84.663
4% FCM yield	32	−0.014	−0.172	0.144	0.080	0.862	0.000	30.629	0.485	0.000
Milk composition										
Lactose concentration	40	0.403	0.023	0.784	0.194	0.038	1.067	184.792	<0.001	78.354
Fat concentration	46	−0.629	−0.923	−0.336	0.150	<0.001	0.670	155.776	<0.001	70.470
Protein concentration	49	−0.361	−0.627	−0.094	0.136	0.080	0.524	143.962	<0.001	66.658

DMI, dry matter intake; BW, body weight; BCS, body condition score; FCM, fat corrected milk.

**Table 4:** Effects of feed extrusion on digestibility and ruminal fermentation

Variables	NC	Estimate	Lower bound	Upper bound	Std. error	p-value	τ^2^	Q	Het. p-value	I^2^
Digestibility										
DM	42	0.252	0.017	0.487	0.120	0.036	0.240	75.183	0.001	45.466
OM	52	0.081	−0.134	0.296	0.110	0.461	0.249	92.553	<0.001	44.896
CP	52	0.604	0.254	0.953	0.178	0.001	0.843	159.292	<0.001	67.983
NDF	31	−0.085	−0.460	0.290	0.191	0.657	0.631	86.402	<0.001	65.278
ADF	18	−0.014	−0.231	0.202	0.111	0.897	0.000	13.923	0.673	0.000
Ruminal fermentation										
Total VFA	33	0.240	−0.205	0.685	0.227	0.291	1.110	117.214	<0.001	72.700
Acetate	49	0.022	−0.233	0.277	0.130	0.866	0.455	118.925	<0.001	59.638
Propionate	49	−0.122	−0.303	0.058	0.092	0.185	0.093	63.097	0.071	23.926
Butyrate	47	0.318	0.066	0.570	0.129	0.014	0.400	105.141	<0.001	56.249
Iso-butyrate	31	−0.438	−0.844	−0.033	0.207	0.034	0.856	98.266	<0.001	69.471
Iso-valerate	34	−0.572	−0.883	−0.261	0.159	<0.001	0.405	67.831	<0.001	51.350
Valerate	34	0.680	0.210	1.150	0.240	0.005	1.422	143.544	<0.001	77.010

DM, dry matter; OM, organic matter; CP, crude protein; NDF, neutral detergent fiber; ADF, acid detergent fiber; VFA, volatile fatty acid.
